# Association between human leukocyte antigen class II and pulmonary tuberculosis due to mycobacterium tuberculosis in Uganda

**DOI:** 10.1186/s12879-016-1346-0

**Published:** 2016-01-23

**Authors:** Dan Wamala, Helen Koyokoyo Buteme, Samuel Kirimunda, Gunilla Kallenius, Moses Joloba

**Affiliations:** 1 Department of Pathology, Mulago Hospital and Makerere University, Kampala, Uganda; 2 Department of Clinical Science and Education, Södersjukhuset, Karolinska Institutet, Stockholm, Sweden; 3 Department of Medical Microbiology, School of Biomedical Sciences, College of Health Sciences, Makerere University, Kampala, Uganda

**Keywords:** Human leukocyte antigen, Major histocompatibility complex, Polymerase chain reaction, Tuberculosis, Uganda genotype

## Abstract

**Background:**

*Mycobacterium tuberculosis* (Mtb) is reported to infect about a third of the world’s population but only 10 % are thought to develop active tuberculosis (TB) disease. Host immunity regulated by human leukocyte antigens (HLA) is an important determinant of the outcome of the disease. Here we investigate HLA class II gene polymorphisms in susceptibility to TB, and whether particular HLA class II alleles were associated with TB in Uganda.

**Methods:**

HIV negative patients with pulmonary TB (*n* = 43) and genetically related healthy household controls (*n* = 42) were typed for their HLA II class alleles using polymerase chain reaction sequence specific primer amplification.

**Results:**

The HLA-DQB1*03:03 allele was significantly less frequent in patients compared to healthy controls (10 % in controls versus 0 % in patients, *p* = 0.003). After correction for multiple comparisons the difference remained significant (*p* = 0.018).

**Conclusions:**

Our results suggest that the HLA-DQB1*03:03 allele may be associated with resistance to TB.

**Electronic supplementary material:**

The online version of this article (doi:10.1186/s12879-016-1346-0) contains supplementary material, which is available to authorized users.

## Background

Tuberculosis (TB) remains a major global cause of morbidity, second only to HIV as a single leading infectious cause of death worldwide [1], and estimated to have caused 8.6 million new infections and 1.3 million deaths in 2012 [2]. In Uganda, the annual incidence of TB is estimated to be 330 cases of all forms and 136 new smear positive cases per 100,000 inhabitants [3]. Uganda ranks 16th among the 22 TB high burden countries [4]. In Uganda the emerging Uganda genotype of *Mycobacterium tuberculosis* (Mtb) is the prevalent cause of pulmonary TB, and accounts for up to 70 % of isolates [5].

**Table 1:** Observed numbers and percentages of human leukocyte antigen Class II HLA-DRB and -DQB antigens in pulmonary TB patients compared to healthy controls for alleles where either the patients or the controls have frequencies > 10 %

HLA Allele	Total count	Pulmonary TB patients		Controls		OR (95 % CI)^b^	p- value^c^	p- value^a,c^
		(*N* = 43)		(*N* = 42)				
		No.	%	No.	%			
DRB1*13:01	33	16	19	17	20	0.9 (0.4–1.9)	0.848	1.000
DQB1*03:03	8	0	0	8	10		0.003	0.018
DQB1*02:01	33	17	20	16	19	1.0 (0.5–2.2)	1.000	1.000
DQB1*03:01	24	15	17	9	11	1.8 (0.7–4.3)	0.272	0.816
DQB1*05:01	44	24	28	20	24	1.2 (0.6–2.5)	0.601	1.000
DQB1*06:01	35	17	20	18	21	0.97 (0.4–1.9)	0.851	1.000

^a^Adjusted with Benjamini & Hochberg (FDR) for 6 test

^b^calculated for alleles where both frequencies > 0 %

^c^Fisher Exact Test

Infection with Mtb is estimated to occur in approximately one third of the world’s population, but 10 % of infected immune competent individuals develop clinically active disease during their lifetime [6]. The outcome of TB infection may depend on host genetic factors, as well as environmental and bacterial factors [7]. Host factors associated with TB pathogenesis are complex and a number of genes contribute to initiation and orchestration of the immune response to TB [8]. Evidence for host determined susceptibility to TB emanates from twin studies that showed significant differences in morbidity between monozygotic and dizygotic twins, [9, 10], genome wide linkage studies [11, 12] and case-control association studies [13].

The human leukocyte antigen (HLA) gene family, i.e., the major histocompatibility complex (MHC) in humans, plays an important role in immune modulation and is essential in initiating an efficient cell mediated immune response [14, 15].

The HLA system is highly polymorphic due to selective influence by infectious diseases, and HLA polymorphisms may influence antigen presentation specificity by modifying peptide binding motifs. Increased binding of the pathogen peptide to binding motifs of the MHC leads to enhanced immunogenicity compared to weak MHC-peptide binding [16].

HLA class II molecules are expressed by antigen-presenting cells, and lymphocytes reactive to class II molecules express CD4 and are often helper T cells. Several studies on the role of HLA II alleles in conferring resistance or susceptibility to TB have been done [17–20] producing conflicting results.

Using polymerase chain reaction amplification with sequence specific primers (PCR-SSP), we here investigate the frequency of HLA Class II alleles in TB patients and healthy controls in Uganda to evaluate whether particular HLA II alleles were associated with susceptibility or resistance to Mtb and in particular to the Mtb Uganda genotype.

## Methods

The study was reviewed and granted ethical approval by Makerere University School of Medicine Internal review Board and Uganda National Council of Science and Technology. Written informed consent to obtain human blood and sputum samples as well as to use isolates from the sputum samples for studies was obtained from all enrolled study participants or their legal guardians.

### Study design

This was a family -based case- control study conducted between 2011 and 2013 at Mulago Teaching and National Referral Hospital (MTNRH), Kampala TB clinic, aimed at determining HLA class II (HLA-DR and -DQ) alleles that confer susceptibility and resistance to TB in general and *Mtb* Uganda genotype in particular.

### Study population

Forty-three HIV negative ethnic African patients with pulmonary TB referred to MTNRH, Kampala TB Clinic were included in the study. In all cases the diagnosis of TB was confirmed with light microscopy that revealed acid fast bacilli in sputum after staining with Ziehl Neelsen stain, and by positive Mtb culture. Forty-two healthy, ethnically and geographically matched controls that were the patients’ biological first degree relatives and living in the same household for 6 or more months but had no pulmonary TB were recruited as controls. The health status of the controls was assessed by eliciting a pertinent medical history and performing general and systemic medical examinations to clinically exclude TB. Control subjects were excluded if they i) had a previous history suggestive of TB ii) previous diagnostic evaluation suggestive of TB iii) prior treatment suggestive of TB iv) symptoms and signs suggestive of TB (cough > 3 weeks, weight loss, dyspnoea, evening fever, night sweats or haemoptysis).

Early morning sputum was collected from each patient. Four ml of peripheral blood were collected from each study subject (pulmonary TB patients and controls) into EDTA vacutainers and stored at −80 °C until used.

### Extraction of DNA

Peripheral venous samples (4 ml) were collected in EDTA vacutainers and stored at −80 °C. 200 μl of whole blood was transferred into a micro-centrifuge tube and DNA extracted using an Epicentre MasterPure DNA purification kit according to the kit manufacturer’s instructions.

### DNA quantification

The DNA quantity was optimized using a GeneQuest (Model Number CE2302), as per the manufacturer’s instructions and confirmed per extraction batch by running a gel with a standard marker.

### HLA allele typing

To analyze for the presence of a given allele, flanking sequences were amplified using alleleic primers based on a sequence-specific oligonucleotide primers (SSP) principle. This was done using the One lambda Micro SSP DNA Typing kit according to the kit manufacturer’s instructions. DNA was amplified by polymerase chain reaction (PCR) for HLA- DR and DQ alleles using sequence specific oligonucleotide primers (PCR-SSP) following the method previously described [21, 22] using an MJ-96 well thermocycler PTC 200.

### Detection of allele amplification

The amplicons were detected by electrophoresis on 2.5 % agarose gels and their patterns visualized under UV after ethidium bromide staining as previously described [22]. The Gel was photographed using a Bioimager- UV transilluminator system (Upland, California).

Typing results were interpreted using the One lambda Micro SSP worksheet provided along with the trays. An internal control band (slower-migrating) was always visible in negative wells (except in the negative control well) as a control for successful amplification. The internal control band was weak or absent in positive wells.

A faster-migrating, positive typing band was observed on the electrophoresis gel when a specific HLA gene was amplified during PCR which indicated a positive test result in a given well. The pattern of positive wells was matched with the information on the Micro SSP worksheet to obtain the HLA type of the DNA sample.

### Genotyping of the Mtb strains

Sputum was homogenized by digestion and decontaminated using the standard N-acetyl-L-cysteine (NALC)-Sodium hydroxide/Sodium citrate method [23] and the resulting sediment was used for making smears and culture on solid Lowenstein Jensen medium and the BACTEC MGIT 960 (Becton Dickinson Diagnostic Systems, Sparks, Md).

DNA was extracted from growth using standard protocols [24] (Reagents from Sigma life Science, USA). Capilia TB assay (TAUN, Numazu, Japan), based on lateral flow immunochromatographic detection of a protein which is highly specific for MTB complex(MPB64) was used to differentiate *M. tuberculosis* complex isolates from non-tuberculous mycobacteria [25].

### Bacterial DNA quantification

DNA was quantified to ascertain its presence and quality using agarose gel electrophoresis and bio image visualization.

### Spoligotyping

All Mtb complex strains were analyzed by spoligotyping using standard protocols [26] (reagents from Ocimum Biosolution) and assigned specific spoligotype nomenclature (SIT) according to the SITVITWEB database [27]. The Uganda genotype is characterized by a spoligotype pattern lacking spacers 40, or both 40 and 43 as previously described [28].

### Region of difference (RD) analysis

All isolates were analyzed for a deletion at the RD724 locus, which is specific for the Uganda genotype as previously described [5].

### Statistical analysis

The research subjects’ Bio data and HLA-DRB and HLA-DQB genotypic frequencies were entered into excel and exported to R v 3.1.3 for analysis.

The number of HLA-DRB1 and HLA-DQB1 genotypes in patients and controls were determined by direct counting. The HLA-DRB and HLA-DQB frequencies were calculated using the following formula: n/Nx2 × 100, where n is the number of alleles found positive for a particular genotype and N is the total number of individuals in each group i.e., pulmonary TB patients and healthy controls. The individual HLA difference in frequency comparisons between TB patients and healthy controls, was assessed using Fisher’s exact test for alleles where either the patients or the controls had an allele frequency > 10 % and the odds ratios (OR) and 95 % confidence intervals (CI) were presented. A p value ≤ 0.05 was considered statistically significant. The p-values were adjusted for multiple comparisons using the Benjamini and Hochberg method.

## Results

The study included 43 TB patients (17 women and 26 men) and 42 control subjects (17 women and 25 men). The mean age of the patients was 27.7 years (range 13–70 years) and the mean age of the controls was 30 years (range 3–57 years). Mtb isolates from 32 TB patients were genotyped, 15 were Uganda genotype and 17 non-Uganda genotype.

The frequencies of each DRB and DQB allele are shown in Additional file 1: Table S1. The most frequent alleles in both the patient and the control groups were DRB1*13:01 (19 and 20 % respectively), DQB1*06:01 (20 and 21 %), DQB1*05:01 (28 and 24 %) and DQB1*02:01 (20 and 19 %) as shown in Table 1. There was no significant difference in the frequency of any HLA-DRB allele between the patients and the controls.

Among the HLA-DQB alleles derived from the 42 controls there were 8 DQB1*03:03 alleles, whereas this allele was not found among the alleles from the 43 TB patients (*p* = 0.003.

No significant differences in allelic frequency of HLA Class II DR and DQ alleles were seen between alleles from patients infected with Mtb Uganda genotype and patients with Mtb Non-Uganda genotypes.

## Discussion

In this study, we analyzed the distribution of HLA-DRB and HLA-DQB alleles in 43 TB patients and 42 healthy controls. We found no HLA class II allele associated with susceptibility to TB. This is in agreement with previous genome-wide scan studies in South Africa and Gambia that did not identify any single major TB-susceptibility gene among Africans [29].

By contrast we found a negative correlation between the HLA- DQB1*03:03 allele and susceptibility to TB (*p* = 0.003) indicating that this allele may confer protection against TB in the study population. After correction for multiple testing, the difference remained statistically significant (*p* = 0.018).

There are now numerous studies from various geographical and ethnic settings on the relation between HLA and TB, and several HLA loci and/or alleles have been associated with both susceptibility and resistance to TB [30]. The association between HLA class II alleles and TB has been studied in different populations with conflicting results [17–20, 31–33]. However, variations in typing methods, small sample size and genetic heterogeneity in the populations studied, both in terms of ethnicity and disease manifestation, makes general conclusions on the role of specific alleles difficult. Early studies were performed by serological tests [30] and need to be confirmed by genetic methods like gene sequencing or PCR-SSP typing to precisely identify the alleles involved.

Studies done in Iran [34] and Thailand [35] found DQB alleles to be associated with susceptibility and or resistance to TB. In a study to analyze HLA-DRB1, DQA1 and DQB1 allelic polymorphism in Iranian patients with pulmonary TB, HLA-DRB1*07 and HLA-DQA1*0101 alleles apparently conferred susceptibility to TB while HLA-DQA*0301 and HLA-DQA*0501 conferred protection against this infection [34]. In Thailand the frequency of DQA1*0601 and DQB1*0301 were decreased in TB patients [35].

In India DRB1∗15 and DRB1∗16, were reported to confer susceptibility to TB in some communities [36] but there was no association in others [17]. Studies conducted on South Indian patients showed no association of HLA-DR and HLA-DQ genes with pulmonary TB. Studies conducted in Indonesia revealed a significant association of DRB1*1202 with pulmonary TB [32], while HLA-DRB1*0212 and HLA-DRB1*16 were associated with resistance to TB in Indonesia and India respectively [32, 33]. HLA-DRB1∗13 alleles apparently conferred resistance to TB in a Polish population [17].

Allele frequencies vary between different ethnic groups and geographical populations [37, 38]. Studies previously done on human HLA allele distribution demonstrated that populations of the same ethnic origin, exposed to similar environmental conditions and sharing a common spectrum of pathogenic exposure tend to have homogenous HLA frequencies [39]. Thus the family based controls used in this study are appropriate in an urban ethnically heterogenous population to reduce confounding factors such as race, ethnicity and genetic background [40, 41].

In our study population DQB1*06:01 and DQB1*05:01, DQB1*02:01 and DRB1*13:01 were the most prevalent alleles but were neither associated with susceptibility nor resistance to TB infection. This is in agreement with the observation that pathogens tend to adapt to the most frequent MHC alleles and rare alleles have selective advantages as perpetual host-pathogen interaction may result in adaptive genetic changes in both the pathogens and host clusters [42]. This may explain why the most frequent alleles in this study population did not confer protection against TB.

In most studies the HLA-DQB1*03:03 allele is of relatively low frequency [34, 38, 43–45], or completely absent [46, 47]. Infection episodes in a population usually result in the emergence of HLA class II alleles through gene mutations or conversion that enable the mounting of an effective immune response to clear the infection [48].

It is interesting to note that the HLA-DQB1*03:03 allele may have a specific role to play in TB pathogenesis. The Mtb culture filtrate protein (CFP) 10 is a potent T cell antigen, and peptide antigens from CFP10 were found to be recognized by CD4^+^ in the context of, among others, DQB1*03, and in particular HLA-DQB1*03:03 [49].

No DQ or DR alleles were significantly associated with Mtb Uganda genotype. However the material was too small to draw any conclusions. Further studies should explore the potential role of HLA-DQB1*03:03 in protection against infections with strains of various genotypes, including the Uganda genotype. A previous study in South Africa demonstrates an association between human HLA types and specific Mtb genotypes and shows that both host and pathogen genetics are important in the development of TB [50]. Strains from a specific lineage may be selected by a human population within a defined geographical background [51].

The findings of this study have implications in product formulation as the discovery of vaccine epitopes that induce protective responses in a particular community is crucial in the development of a new-epitope based protective vaccine.

A major limitation of this study is that it is most likely statistically underpowered due to the small sample size. Possible associations between various HLA-DRB and -DQB alleles and TB may have been missed.

## Conclusions

The HLA-DBQ*03:03 allele was absent in the TB patients and therefore this allele appeared to be associated with resistance to TB. This finding merits further exploration in a larger population. A potential association of HLA Class II DR or DQ genes with TB due to Mtb Uganda genotype could not be ascertained due to lack of statistical power.

## Additional file

Additional file 1: Table S1. Observed numbers and percentages of human leukocyte antigen Class II HLA-DRB and -DQB antigens in pulmonary TB patients compared to healthy controls. Description of data: Table comparing the observed numbers and the percentages of HLA Class II HLA-DRB and -DQB antigens in pulmonary TB patients and healthy controls. (XLS 32 kb)

## Abbreviations

CDC: centers for disease control and prevention
CI: confidence interval
EDTA: ethylenediaminetetraacetic acid
HLA: human leukocyte antigen
MHC: major histocompatibility complex
OR: odds ratio
PCR: polymerase chain reaction
TB: tuberculosis
